# Neurophysiological, Neuroimaging, and Neuropsychological Predictors of Human Alcoholism and Risk

**DOI:** 10.3390/bs13100790

**Published:** 2023-09-22

**Authors:** Chella Kamarajan

**Affiliations:** Department of Psychiatry and Behavioral Sciences, SUNY Downstate Health Sciences University, Brooklyn, NY 11203, USA; chella.kamarajan@downstate.edu

## Abstract

Over the last several decades, both brain electrophysiological measurements, such as electroencephalogram (EEG) and event-related potentials/oscillations (ERPs/EROs), and neuroimaging measures have immensely contributed to our understanding of neural mechanisms underlying various psychiatric disorders, including alcohol use disorder (AUD). This Special Issue was launched to invite research and review articles that explore the utility of these neural measures to determine the effects of alcohol use (e.g., regular drinking, social drinking, binge/heavy drinking, and chronic drinking) on brain structure and function and/or to predict risk for developing AUD and other outcomes (e.g., other drug use and externalizing/internalizing traits) across various demographic characteristics (age, gender, ethnicity, etc.). We received seven scholarly articles, each dealing with specialized topics, which contribute to enhancing our understanding of the brain mechanisms underlying AUD and its risk. The titles of the contributing articles are: (i) Random Forest Classification of Alcohol Use Disorder Using EEG Source Functional Connectivity, Neuropsychological Functioning, and Impulsivity Measures; (ii) Delta Event-Related Oscillations Are Related to a History of Extreme Binge Drinking in Adolescence and Lifetime Suicide Risk; (iii) Alcohol Use and Prefrontal Cortex Volume Trajectories in Young Adults with Mood Disorders and Associated Clinical Outcomes; (iv) Statistical Nonparametric fMRI Maps in the Analysis of Response Inhibition in Abstinent Individuals with History of Alcohol Use Disorder; (v) Differentiating Individuals with and without Alcohol Use Disorder Using Resting-State fMRI Functional Connectivity of Reward Network, Neuropsychological Performance, and Impulsivity Measures; (vi) Epigenetic Effects in HPA Axis Genes Associated with Cortical Thickness, ERP Components and SUD Outcome; and (vii) Predicting Alcohol-Related Memory Problems in Older Adults: A Machine Learning Study with Multi-Domain Features. This Special Issue contains a range of useful topics, covering the utility of EEG, MRI, neuropsychology, epigenetics, environmental, behavioral, and clinical measures related to outcomes, and biological risks related to AUD, which will be useful to alcohol researchers around the world.

## 1. Introduction

We are delighted to present this Special Issue of *Sustainability* entitled “Neurophysiological, Neuroimaging, and Neuropsychological Predictors of Human Alcoholism and Risk”. Over the last several decades, neurophysiological methods, such as electroencephalogram (EEG) and event-related potentials/oscillations (ERPs/EROs), as well as neuroimaging methods such as structural/functional magnetic resonance imaging (s/fMRI), have remained the techniques used most frequently to measure and understand human brain function and disorders. This Special Issue was launched to invite and publish articles that explore the utility of these neural measures to determine the effects of alcohol use (e.g., regular drinking, social drinking, binge/heavy drinking, and chronic drinking) on brain structure and function and/or to predict risk for developing AUD and other related outcomes (e.g., externalizing behaviors and disorders). As expected, the Issue was successful in attracting publications on different topics concerning electrophysiological and neuroimaging methods. The articles in this Special Issue deal with the following topics: (i) differentiating AUD individuals from healthy controls using functional connectivity derived from either EEG (Contribution 1); (ii) use of ERO measures to understand binge drinking in adolescence and lifetime suicide risk (Contribution 2); (iii) associations between alcohol use and PFC structural trajectories in young adults with a mood disorder compared to typically developing peers (Contribution 3); (iv) use of nonparametric permutation-based fMRI analysis to elucidate brain regions and networks associated with response inhibition in abstinent AUD and control participants (Contribution 4); (v) differentiating AUD individuals from healthy controls using functional connectivity derived from fMRI functional connectivity across the reward network, together with measures of neuropsychological functioning and impulsivity (Contribution 5); (vi) how early life adversity in childhood can impact the methylation of the *CRHR1* gene with implications for brain development as seen in cortical thickness and ERP signals emanating from particular brain regions, which are associated with risk for developing AUD (Contribution 6); and (vii) predicting alcohol-related memory problems in older adults using resting-state EEG source connectivity and multi-domain features (i.e., neuropsychological, genetic, behavioral, and clinical) in a machine learning model (Contribution 7). Each of these articles has contributed to enhancing our understanding of neurobiological, cognitive, and behavioral factors underlying AUD and its risk. 

## 2. Human Neurophysiological and Neuroimaging Research on Alcohol Use Disorder

The research papers published in this Special Issue have enormously contributed to enhancing our understanding of brain mechanisms underlying AUD and the biological risk factors for developing the disorder. In “Random Forest Classification of Alcohol Use Disorder Using EEG Source Functional Connectivity, Neuropsychological Functioning, and Impulsivity Measures (Contribution 1)”, Kamarajan et al., used a random forest classification algorithm to differentiate AUD individuals (*n* = 30) from healthy controls (*n* = 30) by using EEG–source functional connectivity (derived using eLORETA software), along with neuropsychological performance and impulsivity measures, as predictors. They reported that the AUD individuals showed a predominant pattern of hyperconnectivity (in 25 of 29 default-mode network connections, which included 13 hippocampal-cortical connections), suggesting altered network functioning indicative of neural hyperexcitability and impulsivity. The AUD group also showed poorer memory performance on the VST task and increased impulsivity compared to control participants. 

In “Delta Event-Related Oscillations Are Related to a History of Extreme Binge Drinking in Adolescence and Lifetime Suicide Risk (Contribution 2)”, Ehlers et al., investigated early alcohol exposure, as well as other risk behaviors, such as suicidal thoughts and actions, by using ERO signals related to emotion processing in young adults (age 18–30 years) of American Indian (*n* = 479) and Mexican American (*n* = 705) ancestries. The results showed that extreme adolescent binge drinking was associated with increased delta ERO activity and decreased phase locking, particularly in the parietal region, suggesting that ERO measures may represent potential biomarkers of adolescent extreme binge drinking and risk for suicidal behaviors. 

In “Alcohol Use and Prefrontal Cortex Volume Trajectories in Young Adults with Mood Disorders and Associated Clinical Outcomes (Contribution 3)”, Kirsch et al., investigated associations between alcohol use and trajectories of prefrontal cortical (PFC) volume in young adults with a mood disorder compared to typically developing peers. The results confirmed that greater alcohol use was prospectively associated with decreased PFC volume in participants with mood disorders, but not in the typically developing comparison participants, indicating possible prefrontal involvement in both mood disorders and alcohol use.

In the fMRI study “Statistical Nonparametric fMRI Maps in the Analysis of Response Inhibition in Abstinent Individuals with History of Alcohol Use Disorder (Contribution 4)”, Pandey et al., attempted to confirm the brain regions and circuits associated with response inhibition and to determine whether these regions were differentially activated in abstinent AUD compared to control participants by using a nonparametric permutation-based fMRI processing. It was found that lower activation in regions associated with the inhibitory control and ventral attentional network distinguished abstinent individuals with AUD from the control participants, implicating an altered cognitive control network in abstinent individuals with past AUD diagnosis. 

In the machine learning study “Differentiating Individuals with and without Alcohol Use Disorder Using Resting-State fMRI Functional Connectivity of Reward Network, Neuropsychological Performance, and Impulsivity Measures (Contribution 5)”, Kamarajan et al., examined functional connectivity across the reward network regions derived from resting-state fMRI on the same set of AUD and control participants (*n* = 60) and found that AUD participants showed hypoconnectivity in nine connections across thirteen regions, as well as hyperconnectivity in three connections involving six regions of the reward network, confirming the previously reported findings of altered reward processing in AUD individuals.

In “Epigenetic Effects in HPA Axis Genes Associated with Cortical Thickness, ERP Components and SUD Outcome (Contribution 6)”, Hill et al., used multimodal measures, such as MRI, ERP, epigenetic marker, and early life adversity in childhood, and found that visual P300 amplitude at the parietal region and cortical thickness of the left lateral orbitofrontal region were significantly related to AUD risk status. Additionally, orbitofrontal cortical thickness was negatively correlated with the methylation status of the *CRHR1* gene as well as with childhood stress scores, which in turn was associated with P300 amplitude recorded in childhood. This important study suggested that early life adversity in childhood can impact the methylation of the *CRHR1* gene and brain development reflected by cortical thickness and brain electrophysiological signals.

Lastly, in the study on predictive modeling “Predicting Alcohol-Related Memory Problems in Older Adults: A Machine Learning Study with Multi-Domain Features (Contribution 7)”, Kamarajan et al., used the resting-state EEG source connectivity of the default-mode network, which was collected ~18 years ago, together with other features (i.e., neuropsychological, genetic, behavioral, and clinical), in a random forest classification model to classify a group of 94 individuals (ages 50–81 years) with alcohol-induced memory problems against an equal number of matched control participants who did not have memory problems. It was found that the memory group manifested a predominant pattern of hyperconnectivity across the default mode network regions, including the connections across the hippocampal hub regions. On the other hand, a few of the connections involving the anterior cingulate cortex were found to be hypoconnected. Other significant predictors of memory problems included polygenic risk scores for AUD, alcohol consumption and related health consequences, elevated neuroticism and increased harm avoidance, and fewer positive “uplift” life events. The study outlined the importance of utilizing multidomain features to predict alcohol-related consequences that could arise about two decades later in life.

Overall, all of these studies, by investigating brain measures and other biological and behavioral correlates, have yielded important findings that provide additional insights into understanding the risks and consequences of AUD. These innovative findings have added to the existing knowledge base in the field of addiction, and therefore these articles can be a reference source for biomedical researchers as well as clinicians. As the Guest Editor of this Special Issue, I greatly appreciate the excellent contributions of the authors. I also express my gratitude to the reviewers for their valuable time and expertise, which were critical for ensuring the quality of this Special Issue and the journal.

## 3. Future Research Directions in Alcohol Use Disorder

While the studies published in this Special Issue have made valuable scientific contributions to the field of alcohol research, there are some limitations, as mentioned in the articles. In view of these potential limitations, suggestions for future research to improve the scope and methodology might be useful, as the reported findings may not be fully conclusive, generalizable, or replicable. First, sample sizes in most “data-based” research must be increased using methods that include the merging of datasets from multiple studies or consortia. Second, meta-analytic studies may be helpful to confirm the pre-existing hypotheses related to specific neural effects on AUD and risk. Third, longitudinal studies with a large sample size are required to draw causal inferences, which are essential for ascertaining the nature of the relationship between the predictors and clinical outcomes. Fourth, studies should use multimodal datasets (e.g., neural, cognitive, behavioral, and clinical) to predict clinical outcomes related to alcohol and other substance use disorders in order to answer many unresolved questions in the field of addiction. Finally, the issue of the generalizability and specificity of findings should be addressed by conducting replication and validation studies using various samples and datasets from across the world.

## 4. Conclusions

As noted above, this Special Issue collates an interesting set of research articles that have explored the antecedents, predictors, and correlates of AUD and biological risk for developing the disorder. It has also dealt with a range of topics that are of great interest to alcohol researchers around the world, covering the utility of EEG, MRI, neuropsychology, epigenetics, environmental, behavioral, and clinical measures, to understand and predict outcomes and risks related to AUD. Special Issues with similar broad topics in the future may be very helpful to the scientific community at large.

## 5. List of Contributions

1. Kamarajan, C.; Ardekani, B.A.; Pandey, A.K.; Chorlian, D.B.; Kinreich, S.; Pandey, G.; Meyers, J.L.; Zhang, J.; Kuang, W.; Stimus, A.T.; et al. Random Forest Classification of Alcohol Use Disorder Using EEG Source Functional Connectivity, Neuropsychological Functioning, and Impulsivity Measures. *Behav. Sci.* **2020**, *10*, 62. https://doi.org/10.3390/bs10030062.
2. Ehlers, C.L.; Wills, D.N.; Karriker-Jaffe, K.J.; Gilder, D.A.; Phillips, E.; Bernert, R.A. Delta Event-Related Oscillations Are Related to a History of Extreme Binge Drinking in Adolescence and Lifetime Suicide Risk. *Behav. Sci.* **2020**, *10*, 154. https://doi.org/10.3390/bs10100154.
3. Kirsch, D.E.; Tretyak, V.; Le, V.; Huffman, A.; Fromme, K.; Strakowski, S.M.; Lippard, E.T.C. Alcohol Use and Prefrontal Cortex Volume Trajectories in Young Adults with Mood Disorders and Associated Clinical Outcomes. *Behav. Sci.* **2022**, *12*, 57. https://doi.org/10.3390/bs12030057.
4. Pandey, A.K.; Ardekani, B.A.; Byrne, K.N.; Kamarajan, C.; Zhang, J.; Pandey, G.; Meyers, J.L.; Kinreich, S.; Chorlian, D.B.; Kuang, W.; et al. Statistical Nonparametric fMRI Maps in the Analysis of Response Inhibition in Abstinent Individuals with History of Alcohol Use Disorder. *Behav. Sci.* **2022**, *12*, 121. https://doi.org/10.3390/bs12050121.
5. Kamarajan, C.; Ardekani, B.A.; Pandey, A.K.; Kinreich, S.; Pandey, G.; Chorlian, D.B.; Meyers, J.L.; Zhang, J.; Bermudez, E.; Kuang, W.; et al. Differentiating Individuals with and without Alcohol Use Disorder Using Resting-State fMRI Functional Connectivity of Reward Network, Neuropsychological Performance, and Impulsivity Measures. *Behav. Sci.* **2022**, *12*, 128. https://doi.org/10.3390/bs12050128.
6. Hill, S.Y.; Wellman, J.L.; Zezza, N.; Steinhauer, S.R.; Sharma, V.; Holmes, B. Epigenetic Effects in HPA Axis Genes Associated with Cortical Thickness, ERP Components and SUD Outcome. *Behav. Sci.* **2022**, *12*, 347. https://doi.org/10.3390/bs12100347.
7. Kamarajan, C.; Pandey, A.K.; Chorlian, D.B.; Meyers, J.L.; Kinreich, S.; Pandey, G.; Subbie-Saenz de Viteri, S.; Zhang, J.; Kuang, W.; Barr, P.B.; et al. Predicting Alcohol-Related Memory Problems in Older Adults: A Machine Learning Study with Multi-Domain Features. *Behav. Sci.* **2023**, *13*, 427. https://doi.org/10.3390/bs13050427.

